# Understanding Parents’ Experiences with Children with Type 1 Diabetes: A Qualitative Inquiry

**DOI:** 10.3390/ijerph19010554

**Published:** 2022-01-04

**Authors:** Justin A. Haegele, Steven K. Holland, Eddie Hill

**Affiliations:** 1 Department of Human Movement Sciences and Center for Movement, Health & Disability, Darden College of Education and Professional Studies, Old Dominion University, Norfolk, VA 23529, USA; jhaegele@odu.edu (J.A.H.); ehill@odu.edu (E.H.); 2 Department of Teacher Education, Faculty of Social Sciences and Education, NTNU—Norwegian University of Science and Technology, 7491 Trondheim, Norway

**Keywords:** diabetes care, family planning, chronic health condition

## Abstract

Parenting is often described as a stress-inducing experience, which can be further complicated or made more stressful and anxiety-inducing when parenting children with chronic conditions such as type 1 diabetes (T1D). The incidence of T1D among children has risen and continues to rise globally, resulting in a need to understand the experiences of parenting children with T1D. The purpose of this qualitative inquiry was to explore the lived experiences, and the meaning ascribed to those experiences, of being a parent of a child with T1D. This qualitative study was conducted through an interpretivist paradigm and includes the experiences of 29 parents (19 mothers and 10 fathers) of 24 children (aged 6 to 15 years) with T1D. Parents, and parent dyads, completed demographic questionnaires and written prompts, and participated in focus group interviews. Three themes were developed from the data, namely, (a) the costs of T1D, (b) the ultimate helicopter mom, and (c) dealing with “being different”. Generally, the participants reported on the direct (e.g., financial and time) and indirect (e.g., family planning) costs associated with parenting children with T1D, their role as a primary provider and anxieties with relinquishing control and dealing with the stigma surrounding a diabetes diagnosis. Unique findings from this study included the impact a T1D diagnosis had on future family planning as parents navigated the fear and uncertainty of having additional children with T1D, as well as the internal conflict parents had with entrusting others to care for their child, especially if they deemed them to be unqualified or unnecessarily stigmatizing or ostracizing their child.

## 1. Understanding Parents’ Experiences with Children with Type 1 Diabetes

As stated by Albanese and colleagues [1], “parenthood, though often joyful, is also an experience rife with stress-inducing challenges” (p. 333). This may be particularly true for parents of children with type 1 diabetes (T1D), a chronic condition often diagnosed during childhood, and one of the most common among youth [2]. On average, the global incidence of new diagnoses among children is 3% to 4% per annum [3], and the prevalence of T1D diagnosis has increased in recent decades [2]. This trend is reflected in the US where the Centers for Disease Control and Prevention [4] reports that from 2002 to 2015, the overall incidence of T1D significantly increased, and, in 2018, approximately 187,000 children and adolescents under 20 years of age had diagnosed T1D.

Parenting children with T1D has been described as a “huge responsibility” [5] (p. 25), which may be particularly challenging and complex [6]. To date, several studies have examined the experiences of parents of children with T1D. These studies, which have discussed mothers’ and fathers’ experiences both separately and together, have explored experiences associated with the diagnosis event, diabetes management, emotional burdens related to finding secondary caregivers, restrictions in physical activity, and parenting in general [5,6,7,8,9,10]. This line of inquiry has highlighted the complexity of particularities associated with parenting children with T1D, as well as psychosocial challenges (e.g., anxiety, depression, stress, and distress) parents can experience when caring for a child with T1D [7,9]. For example, in a study exploring the experiences of mothers with young children with T1D, participants reported needing to be hypervigilant to accomplish daily management tasks while navigating undesirable social interactions with other parents regarding their care for their child [10]. 

Understanding the perceptions and experiences of parents of children with T1D, including their role in T1D management and the meaning they ascribe to that role, can hold great value [8]. Despite growth in this area of inquiry, there is still more to be discovered about the particularities of how one experiences being a parent of a child with T1D. For example, the existing research has largely focused on parenting young children [5,7,10], with few exploring experiences of those parenting adolescents. One example though, from Mellin and colleagues [8], examined the experiences of parents of 30 adolescent girls with T1D, and reported both the negative (e.g., parental stress) and positive influences (e.g., promoting responsibility) of T1D on their families. As such, currently, only a small age range is represented in the extant literature. In addition, while several studies have been conducted that provide descriptive accounts of parenting children with T1D, few explore the meaning and feelings that parents ascribe to these experiences; that is, a better understanding of the lived experiences of parents of children with T1D can help inform service providers, such as diabetes educators, on unique particularities that can shape experiences and act as the focus of interventions. The qualitative research paradigm has been described as an ideal approach to engage in in-depth explorations of unique challenges and factors associated with T1D and parenting children with T1D [2,7,11]. This is echoed by Anderson and Robins [12], who asserted that qualitative research can make an important contribution to deepening our understanding of the experiences of those involved in diabetes and diabetes care. Qualitative studies are particularly important when aiming to improve diabetes education or interventions by first understanding experiences and the meaning of experiences [11].

By focusing on qualitative research, we can explore the meaning ascribed to the experiences of parents of children with T1D and understand salient features that contribute to those meanings and feelings. This focus can provide much needed nuance to our understanding of parenting experiences, yet is particularly absent from studies conducted in the US. A better understanding of the meaning and feelings that parents ascribe to their experiences could help promote more positive approaches to T1D management and contribute to the development of interventions [13]. As such, the purpose of this study was to explore the lived experiences, and the meaning ascribed to those experiences, of being a parent of a child with T1D. 

## 2. Methods

Maher and Coates [14] encourage researchers to acknowledge the philosophical underpinnings that influence methodological decisions made in qualitative research. In this study, an interpretivist research paradigm was adopted, with an emphasis on understanding our participants’ interpretations of their lived experiences as parents of children with T1D. The interpretivist research paradigm is underpinned by a relativist ontology and subjective epistemology, which influenced the research team members’ perspectives and behaviors throughout the research process [15]. 

### 2.1. Participants

A convenience sample of 29 parents (19 mothers, 10 fathers) of children with T1D were recruited to participate in this study. Parents were recruited to participate in a one-day recreation program for children with T1D located in [location anonymized for review]. Each parent reported having just one child with T1D, and an average of 2.58 children overall (range 1–4 children). The 29 parents included 10 parenting dyads who shared children, and therefore, the 29 parents represented a total of 24 children with T1D. Of those 24 children, 13 were girls and 11 were boys, and their ages ranged from 6 to 15 years (M_age_ *=* 10.5 years). Seventeen children were White, four were Hispanic, two were Black, and one was both White and Hispanic. Parents were asked to report their children’s last known hemoglobin A1c (HbA1c) level, and responses ranged from 5.9 to >15. Parents also reported that their children were diagnosed with T1D from three months to seven years (M_years_ *=* 3.015 years) prior to the interview. 

### 2.2. Procedures

The institutional review board at Old Dominion University approved this study prior to the implementation of study procedures. Parents were informed of this study, as well as the opportunity to participate, via email after the enrollment of their child, in the one-day recreation program. Those who were interested in participating in the study were asked to plan to remain at the one-day program to complete data collection while their child participated in activities in a separate area. Interested parents were provided an informed consent document, and those who signed and completed the document were enrolled in the study.

Three data collection methods were used for this study: a demographic questionnaire, a brief written prompt, and semi-structured focus group interviews. First, parents were asked to complete an eight-item demographic survey, which asked them to report: a) how many children they had, b) how many of those children had T1D, c) what their familial role was (e.g., mother and father), d) how old their child with T1D was, e) how many years it had been since their child was diagnosed with T1D, f) their child’s last known HbA1c level, g) their child’s gender, and h) their child’s race/ethnicity. All responses were open-ended, allowing the participants to interpret for themselves what best represented their family and children.

The second source of data was a brief written prompt. After the completion of the demographic survey, participants were asked to respond to the following written prompt: “In the space below, can you describe in your own words what it’s like to be a parent of a child with diabetes and send them to schools?” Data specific to schools were used in a separate analysis and are presented elsewhere. This written prompt was implemented to allow for participants to have a place to privately reflect upon their experiences as a parent, to stimulate thinking about what they might want to share with the other parents, and to record feelings and experiences that they did not want to share with other parents during focus group interviews. Parents were instructed to keep these written prompts throughout the focus group interviews so they could continue to add to what they recorded when the experiences shared by others stimulated further thinking and reflection. 

The final, and primary, source of data for this study was audio-recorded focus group interviews. The 29 participants engaged in one of four focus group interviews, which took place in a private location that was separated from the children and volunteers participating in the recreation program. Interviews included seven, nine, three, and ten parents, and were 63, 49, 50, and 67 min in length, respectively. Prior to the interviews, the interviewer exposed his personal and professional positionalities to the participants, to support transparency during the data collection process. All interviews were facilitated by the lead author and were guided by a semi-structured interview guide that was inspired by the research focus and approach. This interview guide was used as a checklist to ensure that similar lines of questions were followed across groups, while allowing the conversations to drive the order and magnitude of the discussion [16]. Questions were purposely open and expansive, to encourage discussion among participants. The guide was informed by the interpretive research approach underpinning this study, as well as prior research exploring parenting experiences. The guide was initially developed by the first author and reviewed by each member of the research team, as well as a scholar outside of the authorship team with familiarity within this area of inquiry, to ensure relevancy. Among the authorship team members is a faculty member with over 15 years of experience as a service-provider for families with children with T1D, whose experience was drawn upon during the construction and refinement of the interview guide. 

### 2.3. Data Analysis 

Following the interviews, audio files were transcribed verbatim. One research team member acted as the analyst (J.A.H.), and thematically analyzed the data using a three-step process that was both iterative and interpretive. The first step involved data immersion, where the analyst read and re-read each of the prompt responses and focus group interview transcripts multiple times to gain an intimate understanding of the participants’ experiences. Descriptive and exploratory commentary associated with meaningful pieces of data were recorded at this time [17]. Second, the analyst constructed initial codes from the data and commentary that were central to the participants experiences. At this stage, the third author was drawn upon as a critical friend to review and challenge the initial interpretations of the data by the analyst [18]. The final step of the analysis included the combination of the constructed codes into overarching themes. These themes were again reviewed by the third author to ensure that they reflected the participants’ experiences and were in alignment with the purpose of the study [19]. Constructed overarching themes are presented and exemplified as themes.

Several steps were taken in order to support trustworthiness during this study. First, the analysis was conducted by one research team member, who recorded detailed notes in the form of an audit trail, as it is recognized that the background knowledge and experience of the analyst inescapably influences data interpretation. During this process, a second research team member acted as a critical friend, who challenged interpretations of the analyst. Transparency and coherence were supported throughout the research process by describing critical elements thoroughly, including data collection and analysis procedures. Finally, an abundance of quotes are provided throughout the results section, in an effort to allow readers to review interpretations and safeguard against themes echoing existing findings. 

## 3. Findings

Results are presented as three themes that depict the experiences of our participants as parents of children with T1D: a) the costs of T1D, b) the ultimate helicopter mom, and c) dealing with “being different”. 

### 3.1. The Costs of T1D

The first theme depicts the parents’ reflections on the costs associated with T1D, a prominent feature in the participants’ narratives when describing their experiences. Financial and time-related considerations were direct costs that were often discussed interrelatedly. These costs largely centered on parents’ experiences with navigating insurance companies for financial expenses associated with blood glucose monitors. Illustrating this, participants reflected that blood glucose monitors are “just too much money, just too expensive” but also that “insurance is so expensive, like $350.00 per month”. As such, participants appeared to be at a crossroads, believing they needed insurance to afford necessary equipment, but also finding insurance expenses for those monitors to be prohibitive. The time spent navigating insurance companies further complicated these issues. For example, one Focus Group 3 participant described that:

We had a huge issue with pods (e.g., Omnipod), because we have to change her pods every two days instead of every three, and the insurance company wouldn’t approve it. I had to get a special form filled out by our doctor, and when that was complete the insurance company dropped the company we were getting the pods from. This was a two-month process, and just now they were telling me this. So that was that…insurance companies are terrible.

Following this participant’s description, another parent in Focus Group 3 added that insurance companies are frustrating “especially when you work all day, and then you come home and have like, a half hour to make that phone call before the company closes”. These types of experiences, where participants clearly depicted financial and time-related costs, highlighted their disinterest in dealing with, a general dissatisfaction for, and animosity toward insurance companies.

In addition to financial and time-related costs, other hidden and less objective costs related to family planning were also described among participants. More specifically, several participants described stresses and fears associated with adding additional children to their family after their child received a T1D diagnosis. For example, a Focus Group 2 parent explained:

I’m fearful to have more children, to be quite honest. I have it in the back of my head, whether or not the next child I have would be diabetic or not. We (husband and I) were just having this conversation while walking over here. I think after four years (since diagnosis), there’s nothing that I don’t think I can handle, but I wonder if this is something I want to handle.

This sentiment was echoed from several other participants, who noted that “I understand your fear, because I have (fears) too after (son’s name) and “we’ve been through it too”. This fear was further supported by another parent, who described the fear that her other child has regarding T1D. This parent recalled that her other daughter asks, “I can have diabetes?”, to which the parent stated “I don’t know, I don’t know how to answer her. So, I understand your fear, it’s still there for me. When you have other kids, I still fear it.” Despite another parent’s attempt to ease their child’s fears by stating that “the odds are only slightly increased that other siblings may get (T1D)”, it was clear that this fear and apprehension was a hidden cost, which is perhaps less quantifiable than the financial or time-related costs experienced by the parents in this study. 

### 3.2. Ultimate Helicopter Mom

The second theme depicts the parents’ reflections about the roles that they play in their children’s lives as a primary T1D care provider, and the trust that they struggle with affording their children, and others, with their children’s care. Across each of the interviews, the parents described playing significant and substantial roles in the lives of their children and their T1D care. For example, parents recalled that “I never really drop the ball” (Focus Group 4) when discussing diabetes care, and that:

I’m hovering all day. I’m texting her at school ‘are you treating?’ The way I think about it is that I’m the *ultimate helicopter mom* because we are way more on top of things than most parents, with everything we do all day long. (Focus Group 3)

The need to be involved in their children’s care was further characterized by a parent from Focus Group 1, who shared an instance where she struggled to come to terms with her child self-caring at night. She explained:

You know, she woke me up in the middle of the night the other night, and she said ‘mom, I just wanted you to know that I woke up and my blood sugar was below 90. So, I went ahead and drank some apple juice, and I’m going back to bed.’ I was like ‘I wish you would have told me before, but you did a good job of checking your blood sugar’. She said ‘well, I know if I came down you were going to tell me to do that, so I just did it.’ It’s hard, I just want to make sure that she is safe.

When discussing feelings associated with the roles that they play within their children’s T1D care, the participants reflected upon their frustration and discomfort with the lack of education, experiences, and limited or inadequate preparation of others regarding T1D and helping their child. Interestingly, this included medical professionals, who, parents noted, receive very little education on T1D (Focus Group 2) and may not have the proper equipment on hand to care for their child (Focus Group 3). 

The level of trust that the parents put into others had clear ramifications, as shown by the restrictions on activities that they would allow their children to engage in, particularly activities that the parents would not attend. Sleepover parties were a subject of extensive discussion across focus groups and a point of contention among parents (audit trail). Many parents noted that they simply would not allow their children to sleep at other people’s houses, reflecting that “I don’t feel like there is a need for my daughter to do that’ (Focus Group 3), or “he (daughter’s father) refuses to let her stay anywhere, because he doesn’t want to put that responsibility of taking care of her T1D on anybody” (Focus Group 1). Other parents, however, saw these types of activities as an avenue for others to gain their trust to take care of their child. For example, a Focus Group 1 parent noted that she asks other parents “’are you willing to come over and take a class and then if she’s (daughter) with you, giving her a shot?’ Then, she can stay or go wherever”, whereas a Focus Group 3 parent reflected that other parents “are hyper concerned, and they want to have all the right foods in the house” when her daughter is over. This trust, however, was fragile, and parents noted that once it was broken, it was unlikely to be gained back. A Focus Group 1 parent highlighted this:

For us, we’ve gotten comfortable with certain people. So, there’s people that we absolutely trust, they can handle it. And then, you know, you come across some people who don’t really want to deal with it. Like, one parent once said, ‘we don’t want to deal with it tonight, so maybe she can’t come sleep at our house tonight.’ And then it’s like, ‘how do I let her stay with you again? Because there was that one time you didn’t want to *deal with it* and it’s really not an option. If she comes to your house, it’s not an option, you have to deal with it.’

This parent exemplified how fragile her trust was and continued by noting that her child no longer spent time with that family after this instance. This example was supported by stories from several parents, one of whom proclaimed that she says to parents “My daughter has T1D, are you okay with this?” to which, if they respond no, then “she’s not coming over”.

### 3.3. Dealing with “Being Different”

The final theme constructed based on the parents’ reflections centers on social interactions surrounding the idea of their child “being different”. Across focus groups, parents described that they have “freaked out”, were “frustrated”, and experienced “ups and downs” when attempting to navigate social interactions with other parents who did not understand their children’s T1D-related needs. For many participants, this issue was perpetuated by navigating stigma associated with diabetes, both for them and for their child. For example, one Focus Group 2 participant noted:

I think it’s part of our society. The name diabetes, you know, for a kid that age. It’s in the name. You hear the word diabetes, and it has a kind of stigma, where someone might think ‘Oh man, what’s wrong with you?’ or ‘Is it contagious?’

Stigma associated with T1D was mentioned among several participants, with another suggesting that “it’s just not a good word” and that her daughter “doesn’t *have* diabetes, she was *diagnosed with* diabetes, because, you know, she’s not diabetes”. This sentiment inspired some parents from Focus Group 3 to deconstruct stigma and normalize diabetes, suggesting the need for their children to act as models socially, such as “other kids seeing (my child) showing a pump” because “they (peers) could see and learn from my child, and see what they are feeling”. A conflicting argument, however, was discussed from a Focus Group 1 parent, who noted that her son “doesn’t want to wear a Dexcom right now out of vanity, because he doesn’t want to appear any more different than anybody else”, and reflected that even though reducing stigma is important, it is generally her child that would feel the brunt of social interactions associated with representing T1D.

For the parents, dealing with “being different” manifested most strongly when other adults had opinions about or attempted to restrict their children’s diet. In this regard, participants recalled that “I kind of felt, I don’t want to use the word *judged*, but I felt *judged*” by other adults due to what they allowed their child with T1D to eat. This experience may be best exemplified by a story shared by a Focus Group 2 parent, who recalled an instance during a holiday event with her church:

No one was limited except my son. The assistant director said to him ‘no, no, no, you can only pick one thing to eat right now.’ When (my son) told me, I called (the assistant director) and said that ‘you cannot single him out that way. I have told you over and over that my choice is that if the other kids get to pick three things to eat, (son) does too. I will deal with the blood sugar if there’s a fall out. It’s not tied to you or controlled by diet.’

The frustration expressed here was shared among many parents, who told various stories about being at social or holiday events and having to defend their children’s ability to eat foods and consume beverages that were initially restricted by other parents. The parents echoed that “our kids are no different than any other kid, they can eat whatever they want, they just have to get the insulin”, and one participant noted that “I’m the mom, and I tell her to do whatever the hell you want” based on scenarios in which her daughter is restricted by other adults who make assumptions about T1D. This perspective was perhaps best summarized by a Focus Group 4 parent, who stated that:

I always liked the meme that said *the only thing kids with diabetes can’t eat is poison or cookies made of poison.* I’m like, we don’t do it all the time. But I don’t do it all the time for the non-diabetic kids either. What’s the difference?

## 4. Discussion

The purpose of this study was to explore the lived experiences, and the meaning ascribed to those experiences, of being a parent of a child with T1D. Grounded in an interpretivist qualitative research paradigm, this study elucidates individual particularities of experiences that contribute to a nuanced and deepened understanding of the experience of parenting children with T1D. As such, three themes were constructed to depict the salient particularities and meanings of these parents’ experiences. In the first theme, parents reflected on the costs associated with T1D, including those that were financial or time-related in nature. These costs, which are largely associated with the technology needed for T1D management, are well aligned with research exploring cost considerations for the adoption of diabetes technology from the perspectives of those with T1D and their families [11]. Interestingly, in their prior work, Addala and colleagues [11] describe navigating insurance companies as a significant cost, in that insurance companies took significant time to navigate, dictate T1D management, and set parameters for technology access. These assertions are well aligned with those of participants in our study, who discussed a number of issues associated with insurance companies, such as the amount of time it took to navigate these companies, restrictions in the number of pods they could receive, and how those restrictions were incongruent with the needs of their child. In addition to financial and time-related costs, a unique cost that was described by parents in this study was that related to family planning, where parents expressed being hesitant to have additional children after their child was diagnosed with T1D. Perhaps these concerns are not unfounded, as family history can influence diagnosis, and it has been reported that a sibling to a child with T1D is eight times more likely to also have T1D in comparison to children in the general population [20]. This finding appears to be a unique contribution to the literature, however, and should help inform diabetes educators and other service providers of an important cost experienced by parents of children with T1D.

Findings from the second theme, which center on the parents’ roles within their children’s T1D management, extends prior research focusing on younger children [5,9]; that is, prior studies have demonstrated that parents of young children with T1D have an intense focus on their children’s needs, which can lead to a variety of stresses and anxieties associated with parenting children with T1D [7,8,9]. This led one parent in the current study to refer to herself as the *Ultimate Helicopter Mom*, a fitting colloquialism for parents who take an overprotective or excessive interest in the lives of their children. Despite the necessity for parents to be engaged in their children’s T1D management [2], there may be some unforeseen consequences. For example, a prior phenomenological study demonstrated that teens with T1D who attempted to transition to autonomous self-management were most confident in their ability to self-manage when their parents provided encouragement to take on their own responsibilities [21]. When this support or encouragement is unavailable, however, confusion between whether to self-manage or rely on parents persists, which can lead to family conflicts and poor T1D management [21,22]. Overprotection in T1D management may also hinder youth from engaging in typical social activities, such as those described by the participants in this study, which can cause a social divide that is difficult to overcome in later years. In this study, in accordance with others [2], parents were hesitant to hand over the reins of their children’s T1D to others, including medical professionals or friends, whom they perceived to be unqualified. In this study, however, only the parents’ perspectives were represented, and therefore, we only understand their perceptions of these experiences. Future research focused in understanding the dynamics between parents and children with T1D regarding T1D management and the roles of parents in this management may consider interviewing both parents and children, to explore if opinions about experiences with T1D diverge or converge between these two parties.

The final constructed theme centers on the parents’ experiences with and feelings about their children being perceived as “different”. In this study, parents expressed frustration and “ups and downs” associated with negative social interactions with other parents and associated these interactions with stigma surrounding T1D. Diabetes-related stigma, which has been identified as being a significant yet under-researched area [23], can have significant social and psychological well-being effects for those with T1D and their families. While concerns about stigma have emerged in other works (e.g., [23,24]), the current study is the first, to the best of the authors’ knowledge, in which parents explicitly discussed diabetes-related stigma and how it influenced their behaviors and experiences as parents.

Among the participants, issues with their children “being different” appeared to most strongly manifest in conversations about dieting and food consumption in instances where other adults were making decisions about their children’s food. These conversations were discussed with frustration, perhaps due to the careful preparation that goes into food choices by parents and children with T1D [2], but also as a result of children with T1D being ostracized or isolated from other children without consent from their parents simply due to misinformation about T1D. This is reflected in a prior study from Sullivan-Bolyai and colleagues [5], where fathers reflected on the gulf between how children with and without chronic health conditions are treated, and about the time they spend ensuring that their children are treated like any other child. This is perhaps best illustrated by a parent in our Focus Group 2, who explained her desire for her child to be treated the same as others, and that she will “deal with the blood sugar if there’s a fall out”.

## 5. Conclusions

This interpretative, qualitative study sought to gain a deeper understanding of the lived experiences of parents of children with T1D, and three themes were constructed to depict these experiences. This study had several strengths, including the focus on elucidating a nuanced understanding of the lived experiences of parents of children with T1D. Readers should be cautious, however, when attempting to generalize this information, as parents were recruited from one region of the US, and generalization is typically not the purpose of this type of inquiry. Other limitations that readers should consider when interpreting our results include the size of the sample, the age dispersion of the children, the variability in the time of diagnosis among children, and the naturalistic formation of the focus groups, all of which could have influenced the way in which the participants discussed their experiences during the focus group interviews. This study is also limited in the understanding of the influence that the particular behaviors or characteristics of children, such as emotional functioning, may have on the experiences of parents. This may be a line of inquiry that would be useful to explore in future work. Generally, the participants reported on the direct (e.g., financial and time) and indirect (e.g., family planning) costs associated with parenting children with T1D, their role as a primary provider and anxieties with relinquishing that control and dealing with being different. Overall, we viewed the depiction of these narratives to be valuable contributions that provide nuanced insight into the lives of parents with T1D, which could be helpful for informing interventions and service provisions. This should include programs that assist parents and children with T1D in transitioning from T1D management control to autonomous self-control, while also helping parents appreciate and embrace their unique family dynamics as parents of children with T1D.
